# Defining Death Anew: Reexamining the Twentieth‐Century Brain Death Debates and the Uniform Determination of Death Act

**DOI:** 10.1002/hast.5005

**Published:** 2025-12-11

**Authors:** Anne E. Clinton

**Keywords:** brain death, defining death, UDDA, clinical ethics, Uniform Law Commission

## Abstract

*In the twentieth century, following the development of advanced life‐support technology, recognition of perimortem states such as brain death challenged traditional definitions of death. A series of debates in the 1960s and 1970s regarding the need to adapt the social and legal frameworks in the face of new medical capabilities culminated in the publication and widespread adoption of the Uniform Determination of Death Act. However, in the interests of uniformity, the drafters of the UDDA compromised its internal conceptual clarity in favor of ensuring that the UDDA was broadly acceptable. A uniform statute proposed by James Bernat and collaborators in the 1980s demonstrates that increased clarity regarding underlying philosophical commitments in a model statute could help to resolve this confusion*.

## Article

Death is a biological process that begins with a clearly living organism and ends with a clearly dead one, but it has many intermediate stages. The reduction of this process into a singular moment of death is an instrument of social functioning. Many practical and legal realities rest on the transformation of a person into a corpse at a particular moment in time: a person becomes a corpse, a patient becomes a decedent, and the rights of an individual and the responsibilities owed to them shift markedly. For most of human history, individuals spent relatively little time in the window between obvious life and obvious death. In the twentieth century, this changed. As advanced life support technology like mechanical ventilators became more and more effective in the mid‐twentieth century, medicine began to recognize new perimortem states. Most prominent of these was *coma depassée*(“beyond coma”), or irreversible coma, which is now called (not without controversy) “brain death.” In 1981, the National Conference of Commissioners on Uniform State Laws (now the Uniform Law Commission) published the Uniform Determination of Death Act, which sought to unify the legal definition of death across jurisdictions. The UDDA was successful in this, but the phenomenon of brain death has remained controversial, particularly with the advancement of life‐support technologies such as extracorporeal membrane oxygenation (ECMO). In 2020, the Uniform Law Commission attempted to revise the UDDA; however, the working group was unable to form a consensus, and the effort was ultimately indefinitely paused in 2023. The legal, philosophical, and medical conversations that arose out of the controversy about how to adapt the concept of death to handle the phenomenon of irreversible coma and careful engagement with the early history of those conversations can provide us with a better understanding of the origins of our current debate and can point toward the steps needed to resolve it.

Here, I will use “irreversible coma” to refer to the syndrome defined by lack of conscious awareness, lack of communication, lack of brain stem reflexes, and lack of spontaneous respiration. I will use “brain death” to refer to the idea that individuals with irreversible, devastating damage to the brain—including, but not always exclusively, the irreversibly comatose—are dead. This definition of brain death lacks specificity in several key areas; this is deliberate. Both the definition of “brain death” and the validity of the concept of brain death were debated during the period under investigation. In addition, I distinguish between the idea of biological death, defined as a complex process ultimately ending with the cessation of unified function of the body, and the idea of social‐practical death, defined as a legal and cultural status that indicates a change in the rights of an individual. After the advent of mechanical ventilation, human intervention could meaningfully interrupt, reverse, and extend the biological dying process. This disrupted the circumstances in which the concept of social‐practical death could be applied. The decision to apply the concept of death no longer rested exclusively on a set of observations regarding the state of the human body; instead, the application of social‐practical death rested on active decision‐making regarding whether to attempt to stall or reverse the dying process.

In the 1960s, the emergence of the phenomenon of irreversible coma complicated the relationship between the concepts of biological and social‐practical death. This led to fierce conversations across the fields of medicine, law, and philosophy about how to realign these two components of the concept of death. This discussion, which played out over the late 1960s, 1970s, and early 1980s, ultimately resulted in the development of the Uniform Determination of Death Act. In 1981, *Defining Death: A Report on the Medical, Legal and Ethical Issues in the Determination of Death*, by the President's Commission for the Study of Ethical Problems in Medicine and Biomedical and Behavioral Research, outlined the reasoning underlying the development of the UDDA.^1^ According to *Defining Death*, one of the most significant goals of the UDDA was to promote interjurisdictional uniformity in the legal definition of death. As a result, the drafters of the UDDA, wishing to avoid sticky philosophical arguments that might undermine broad support for the statute, deliberately avoided taking a stand on fundamental philosophical questions about the relationship between the neurologic and circulatory definitions of death.

The decision by the UDDA drafters to sidestep these questions was not, however, universally considered appropriate. Neurologist and bioethicist James Bernat argued that the UDDA's lack of clarity left concerning gaps in the definition of death. He provided an alternate proposal that would have clarified many of the UDDA's ambiguities. Despite the act's definitional gaps, however, the UDDA was effective in promoting uniformity: it was quickly and broadly adopted, and it is currently the law in thirty‐seven states.^2^ I contend that this deliberate lack of specificity in the UDDA has resulted in ambiguity in our common social and philosophical framework for defining death and that those ambiguities must be addressed in a statutory revision of the UDDA.

## Concept

The existence of the concept of brain death is a product of the technological advances of the 1960s, through which it became possible, for the first time in human history, to maintain cardiopulmonary function artificially in the absence of stimulation from the brain.^3^ In a natural state, the neurological and cardiopulmonary systems have obligate interconnectivity: without the blood supplied by the cardiopulmonary system, the brain dies, and without the stimulation of the brain, the cardiopulmonary system ceases to function. In the absence of technological intervention, the failure of one of these systems will inevitably lead to the failure of the other. Mechanical ventilation, and later innovations such as ECMO, allow for the functioning of the cardiopulmonary system in the absence of stimulation from the brain, creating the possibility of a physical state where the interdependence of these systems is interrupted. Just as technology interrupted these biological connections, so did it interrupt the traditional definitions of death, which did not separate the cessation of cardiopulmonary activity from the cessation of brain function. This resulted in the development of a social phenomenon called “irreversible coma,” a term that was applied to patients who had little to no hope for neurologic recovery but whose bodies could be maintained through artificial cardiopulmonary support.^4^ This resulted in practical consequences: caring for the irreversibly comatose took up limited resources.

In 1968, the Harvard Ad‐Hoc Committee on Brain Death, chaired by neurologist Henry Beecher, published a short but highly impactful paper. The report argued that irreversible coma should be treated as equivalent to circulatory death, and it laid out a set of criteria for determining that a comatose individual was “brain dead,” including absence of brain stem reflexes, lack of spontaneous respiration, and a flat electroencephalogram.^5^ The committee cited practical concerns—namely, the burden of treatment on patients and families and the cost to the health care system—as the primary motivations for shifting the definition of death.^6^

In the late 1960s, there was not a widespread consensus about the appropriate withdrawal of life‐sustaining treatment. The landmark legal case *In re Quinlan*, which established the right of a surrogate decision‐maker to discontinue life‐sustaining treatment, was not decided until 1976.^7^ As a result, there was no consistent accepted method to remove a living comatose patient from artificial cardiopulmonary support. However, if an individual in an irreversible coma were actually dead, then the responsibility to continue life‐sustaining treatment would end. Patients could then be removed from artificial support, at which time the cardiopulmonary system would, in the absence of neurological stimulation, cease to function. The application of the concept of death to irreversible coma thus addressed the practical problem of resource allocation.

Another practical issue that brain death addressed was that of organ transplantation. Organ transplantation began to emerge as a viable treatment in the early 1960s with live‐donor kidney transplants, but most organs were not compatible with live‐donor transplantation. Brain death solved many of the practical issues of transplant. Brain‐dead organ donors did not need to be removed from cardiopulmonary support and allowed to die by circulatory criteria; because they were already dead, organs could be removed directly, dramatically decreasing the time organs were exposed to ischemic conditions and thus improving outcomes for the transplant recipient. Organ transplantation from brain‐dead patients was legally complex, however, and a confrontation in the courts was inevitable.

The first significant legal case involving brain death in the United States came in May of 1968, when doctors at the Medical College of Virginia performed one of the earliest heart transplants. The donor, a Black man named Bruce Tucker, had suffered a cerebral hemorrhage as the result of a fall.^8^ His treating physician thought that the prospects for his recovery were nonexistent.^9^ About twenty hours after he first arrived, the transplant team, headed by Richard Lower, removed his heart and kidneys for transplant.^10^ His family did not give consent for the operation and later sued for wrongful death.^11^ Lower and the other physicians involved with Tucker's case were acquitted in 1972 on the grounds that Tucker was dead prior to the procurement of his organs due to his neurologic state.^12^ This was the first time that cessation of neurologic function was held as a valid legal definition of death in the United States.^13^

*Tucker v. Lower* ^14^ was a herald of the legal confusion—and concerns about that legal confusion—that would come about as a result of the medical community's shift to embrace brain death. In 1970, just two years after the publication of the Harvard criteria and while *Tucker v. Lower* was working its way through the courts, the state of Kansas was the first to pass a statute explicitly recognizing brain death.^15^ This legislation was requested by the transplant program at the University of Kansas Medical Center.^16^ In a letter to the editor published in *the Journal of the American Medical Association* in early 1971 explaining the statute, drafter Loren Taylor cited concern over the results of a 1967 Kansas court case that had affirmed the circulatory definition of death.^17^ He suggested that an explicit change in statutory definitions of death was needed to ensure that physicians were legally protected.^18^ Statutory recognition of brain death as an acceptable definition of death was not universally desired in the medical profession, however. Beecher himself opposed statutory changes, arguing that “when any situation is changing so rapidly … it is not the right time for new and potentially restrictive laws” and that, historically, the intrusion of the “crippling power of the courts” into emerging medical spaces had restricted innovation.^19^ Beecher and Taylor articulated two different lines of thought about the desirability of statutory changes to the definition of death: Beecher felt that the intrusion of the law would have unforeseen consequences for the progress of medical science, whereas Taylor argued that legal intrusion was necessary to protect medical innovation from legal harassment.

In addition to the legal concerns, redefining death raised obvious philosophical questions that required attention. The initial Harvard Ad‐Hoc Committee acknowledged this need both through the inclusion of a theologian and a historian of science among its members and in the text of the report, which observed that “there are moral, ethical, religious, and legal issues. Adequate definition here will prepare the way for better insight into all of these matters.”^20^ The philosophical and ethical concerns at play remained largely implicit in the report, though it did cite the highly influential statements made by Pope Pius XII that “extraordinary means” to prolong survival are not ethically required.^21^

Allowing for the application of the concept of death to the irreversibly comatose solved two problems. First, it allowed for medical teams to handle the practical issue of bedspace by withdrawing cardiopulmonary support in irreversibly comatose patients without the perception that they were hastening patient death or killing a patient. Second, it allowed for the development of transplant programs that respected the dead donor rule. However, the concept was legally dubious without statutory recognition and was not universally desired among physicians. Additionally, redefining a concept as central as death raised major philosophical questions that were not addressed in the formative documents outlining brain death because of their origins in practical concerns. This lack of conceptual foundation would come to haunt brain death as a concept in the coming years, as philosophers and ethicists attempted to create a philosophical substructure for a concept that emerged largely as a result of practical concerns.

## Confusion

The brain death debates of the 1970s were characterized by a diversity of perspectives on multiple axes. The validity of the concept of brain death itself was debated, although, ultimately, a combination of practical pressure and philosophical proponents pushed those who denied the validity of brain death to the fringes. The appropriate extent of the concept was also debated: since the brain has divisible parts, could sections of the brain still be functioning in a person who is brain dead? Finally, how—if at all—should the law respond to these changes? These debates occurred at different levels, from high philosophy to gritty practical details.

One major axis of the debate was the appropriate extent of the concept. There were three major camps: on one end, a reactionary “destructionist” viewpoint held that the only way brain death could be viable was through complete destruction of the entire brain, effectively nullifying the concept. On the other extreme were advocates for neocortical death, who held that death of the cerebral cortex should be sufficient for a brain death diagnosis and thus that the brain stem (and spontaneous respiration) could still be functioning in a brain‐dead individual. The middle ground was composed of advocates for the whole‐brain‐death formulation. Whole‐brain‐death advocates argued that the loss of function of the entire brain, including both neocortex and brain stem, should be required for a brain death diagnosis.

Though the concept of brain death was entrenched by the late 1970s and had strong support in the medical community, its acceptance was not universal.^22^ In a 1979 article, neonatologist and anti‐brain death advocate Paul A. Byrne and collaborators Sean O'Reilly and Paul Quay argued that brain death conflated destruction of the whole brain with loss of function and that, while an individual with a completely destroyed brain could be definitively determined to be dead, the same could not be said for an individual with total loss of brain function.^23^ In this destructionist point of view, loss of function in the absence of destruction could never be definitively proven to be permanent, and as a result, all comatose patients should be regarded as living.^24^ In effect, the destructionist viewpoint narrowed the definition of brain death so profoundly that it ceased to have practical meaning.

In contrast to the extreme conservatism of the destructionist viewpoint, the neocortical death concept radically distanced biological and social‐practical death. Philosopher Robert Veatch promoted neocortical death because, he argued, consciousness was fundamental to the nature of humanity. A permanent loss of consciousness separated an individual from personhood. Thus, an irreversible destruction of consciousness should be equated with death of the person regardless of whether the organism was still living.^25^ Veatch argued that social‐practical death should be defined as “the irreversible loss of that which is essentially significant to the nature of man” and that this “essentially significant” material was consciousness.^26^ Thus, destruction of the neocortex, which was required for consciousness, should be the fundamental definition of death.^27^ For Veatch, the social‐practical concept of death was paramount; biological death was significant only in that it resulted in irreversible loss of consciousness.

Veatch's view was radical, however. In an introductory article for an edition of the *Annals of the New York Academy of Science* focused on brain death, philosopher Richard Roelofs observed that “no one seriously doubts that a decerebrated frog survives his loss of brain tissue, and it is difficult to see … why the human case should be treated differently.”^28^ Roelofs thus suggested that neocortical death represented a concerning philosophical departure from the biological facts. In a reply to Veatch, philosopher Elizabeth Linehan identified a central problem of Veatch's thesis: Veatch contended that it was immoral to treat a corpse as a living human, but Linehan articulated that it was *more* immoral to treat a living human as a corpse.^29^ There was, she contended, a difference between social interaction and consciousness, and it was impossible to know truly that there was no consciousness in a brain that had any remaining functionality. She argued that Veatch inappropriately conflated the ability to interact socially with the capacity for consciousness.^30^ Philosopher Douglas Walton similarly suggested that, while Veatch's position was internally coherent, it rested on shaky foundations: Walton pointed out that medical knowledge had a much higher degree of certainty with regard to the irreversibility of whole‐brain death than of neocortical destruction.^31^ Roelofs's, Walton's, and Linehan's arguments demonstrate that Veatch was unusual in his high degree of comfort with a social‐practical concept of death that was separated from the biological. The neocortical view would thus remain outside the mainstream.

Ultimately, cohesion would come not from a philosophical source but a practical one. In a 1970 reflection on the outcome of the Harvard Committee, Beecher stated that “only a very bold man … would attempt to define death.” For Beecher, the definition of death was distinct from the criteria for death. The definition of death was a philosophical and theological problem, whereas the criteria were a technical problem. There was, therefore, a possibility to change the criteria used to declare death without touching the philosophical core of the concept. Roelofs posited a similar distinction, arguing that the brain death debate was actually composed of two separate issues: a conceptual redefinition of death and a practical redefinition of death. The conceptual redefinition asks if “brain death is a kind of death in the way that a square is a kind of rectangle,” or put another way, whether brain death is equivalent to circulatory death.^32^ The second question is practical—should we *treat* brain death as equivalent to circulatory death? Roelofs argued that, while the first question is philosophically interesting, “when physicians undertake to answer this question[,] they are playing the philosopher's game on the latter's home turf.”^33^ Thus, according to Roelofs, this philosophical question does not need to be answered in order to resolve the practical issues associated with the phenomenon of irreversible coma. Roelofs articulated a viewpoint that would become important in the President's commission's report on defining death—that it was possible to fit brain death into the existing social‐practical framework for death without redefining death conceptually, and thus to avoid engaging in sticky philosophical debates altogether.

The final significant axis of the debate was the appropriate legal response to these conceptual developments, which occurred alongside the philosophical debates. The legal question was broken down into two camps: those who opposed statutory recognition of brain death and those who supported it. Among supporters of statutory change, there were numerous proposals for the form that statutory recognition should take. The anti‐statutory camp argued that, given the rapid evolution of medical technology, caselaw was better positioned to handle the evolution of the concept of death. The pro‐statutory camp argued that, because brain death was a significant shift from the traditional definition of death, preemptive statutory codification was necessary.

The first codification of brain death into law occurred in Kansas in 1970. The statute included two acceptable definitions of death, one defining it by the absence of spontaneous circulatory function and the other by the “absence of spontaneous brain function.”^34^ The section on brain death also explicitly mentioned organ transplant.^35^ The Kansas statute had a mixed response in the medical and legal communities. Medical‐legal scholar Don Harper Mills praised the initiative of the Kansas drafters and noted that “we cannot rely … on the assumption that the courts will necessarily accept medical consensus.”^36^ In contrast, attorney Ian Kennedy concurred with Beecher's earlier concerns regarding the potential for legal interference in medical innovation, arguing that “in an area as fast moving as this, the flexibility to respond to further medical developments must be the keystone of the law.”^37^ The tide toward legislation was strong, however. Following the Kansas statute, other states quickly began debating and passing similar legislation.^38^ Kennedy recognized that statutory codification was inevitable, and in the latter half of his piece, he turned to highlighting specific problems of the Kansas statute—in particular, its explicit focus on transplantation and its dual‐definition format. Kennedy saw this dual definition as a key flaw.^39^

Kennedy was not alone in his criticism. Even those who agreed that a statutory definition was desirable opposed the explicit connection to transplant and the implication in the language of the Kansas statute that there were two separate phenomena of death.^40^ In an influential paper published in 1972, legal scholars Alexander Capron and Leon Kass agreed with Kennedy that the Kansas statute had two major issues: one being that it set a disquieting precedent that standards for death could be different for organ donors and non‐organ donors and the other being the dual definition of death.^41^ Capron and Kass also addressed those who opposed statutory recognition by introducing a four‐level framework of the definition of death, moving from broad to narrow: the “(1) the basic concept or idea; (2) general physiological standards; (3) operational criteria; and (4) specific tests or procedures.”^42^ The Capron‐Kass framework posited, as Beecher had previously, that there was a distinction between the underlying concept of death and the criteria for death (referred to in the Capron‐Kass framework as the “standards”) and that it was possible to legislate the framework without becoming bogged down in the concept.^43^ Capron and Kass proposed their own model, which attempted to resolve the flaws of the Kansas statute and address some of the concerns of the antistatutory group. To resolve the latter concerns, they placed their statute at the level of “general standards,” which they argued would provide uniform legal guidance while allowing for technological innovation.^44^ To address the concerns raised by critics of the Kansas statute, they removed all references to organ donation—the law would apply to all decedents. They also specified the relationship between the circulatory and neurologic criteria for death: according to the Capron‐Kass model, the use of neurologic criteria was acceptable only when artificial support precluded the use of traditional circulatory criteria. With these adjustments, Capron and Kass hoped to create a model statute that would be widely acceptable.

The Capron‐Kass model was adopted by several states but did not become universally accepted.^45^ The American Bar Association published their own proposal in 1975, which was distinguished from the Kansas and Capron‐Kass models by its complete abandonment of circulatory criteria: instead, the phenomenon of death was to be accessed purely via brain death criteria.^46^ This was practically problematic; the vast majority of patients were still (and continue to be) pronounced dead via circulatory criteria. Some states that chose to adopt the ABA model added amendments to clarify that the use of circulatory criteria was also an acceptable standard—but this recreated the issue of the dual phenomena of death of the Kansas statute.^47^ In 1980, there were multiple model statutes in use—the Kansas model, the ABA model, and the Capron‐Kass model—as well as numerous idiosyncratic state statutes.^48^ As a result, there was a high degree of interstate variability in the specific language used in statutory definitions of death, setting the stage for concerns regarding interjurisdictional uniformity for such a consequential concept.

The brain death debates of the 1970s and early 1980s were characterized by a wide spectrum of viewpoints, which nevertheless coalesced around a median position. Views on the appropriate extent of the concept ranged from the radical neocortical position to the highly conservative destructionist viewpoint. The neocortical position advocated by Veatch stabilized the concept of death as a purely social‐practical phenomenon, separating it from biological death. In contrast, the destructionist viewpoint proposed by Byrne and his coauthors did not accept that prior evidence of loss of function could be extended to infer permanent future loss of function. The destructionists would have made brain death a purely theoretical phenomenon, ignoring the practical pressures that had resulted in the emergence of brain death in the first place. The whole‐brain‐death position emerged as a median position on both counts: it kept the social‐practical definition of death in line with biological death but also resolved the practical issues associated with irreversible coma. There was similarly wide variance in statutory proposals. The Kansas proposal included brain death as an entirely alternate definition of death, which separated circulatory and brain death into distinct phenomena. The ABA proposal attempted to unify the phenomenon of death but managed to recreate the issues in the Kansas proposal by ignoring the practical need for circulatory criteria. The Capron‐Kass model resolved the issue of dual phenomena and additionally attempted to avoid the concerns associated with legislating philosophical concepts by legislating criteria (or standards) for death instead. Both the philosophical and legal debates were characterized by a wide range of opinions, but by 1980, median positions had emerged that would prove to be fertile ground for consensus legislation.

## Cohesion

*“An individual who has sustained either (1) irreversible cessation of circulatory and respiratory functions, or (2) irreversible cessation of all functions of the entire brain, including the brain stem, is dead.”* ^49^

*—The Uniform Determination of Death Act, 1980*

At the beginning of 1980s, there was significant variability in the legal status of brain death across the United States. Nonuniformity between jurisdictions was of significant concern from a legal perspective, given the potential for different outcomes in certain unusual cases based on the different language used in the unique statutes.^50^ There was also simply general discomfort with the idea that identical patients could be considered dead in one place but alive in another.^51^ Morris Abram, the chair of the President's Commission for the Study of Ethical Problems in Medicine and Biomedical and Behavioral Research (hereafter, “the President's Commission”) wrote that “uniformity on this matter is a desirable goal. One would expect the same basic rule about who is dead, and who is not, to apply everywhere in the United States.”^52^

As a result, in the early 1980s, there was a concerted effort to bring professional organizations such as the AMA, ABA, and the Uniform Law Commission in line on the subject of a recommended statute for brain death, with federal backing in the form of a report from the President's Commission.^53^ The UDDA was published in August of 1980. The AMA and ABA abandoned their previous proposals in favor of the UDDA. In July of 1981, the President's Commission published *Defining Death: A Report on the Medical, Legal and Ethical Issues in the Determination of Death,* which provides important insight into the rationale behind the UDDA.

This report of the President's Commission explicitly did not seek to explore fundamental philosophical questions, stating that, “while it is valuable to test public policies against basic conceptions of death, philosophical refinement beyond a certain point may not be necessary.”^54^ In fact, the report suggests that further clarification might be counterproductive because it had the potential to undermine broad acceptance of the statute. Alexander Capron, cocreator of the Capron‐Kass model statute, was the executive director of the commission. The priorities he and Kass articulated in their model statute—in particular, the choice to avoid addressing the fundamental philosophical questions raised by redefining death and instead focus on general structural principles and their desire to ensure that death remained a clearly unified phenomenon—remain evident in *Defining Death*.^55^ Like Capron and Kass, this report argues that operating at the level of general physiologic criteria would not interfere with the medical profession's application of new knowledge to adjust the operational and specific criteria but would maintain the core uniformity of the concept of death.^56^ Engaging with the philosophical conceptualization, by contrast, had the potential to “lead down arcane philosophical paths which are at best somewhat removed from practical application.”^57^ The authors of *Defining Death* saw little benefit to engaging with the messy philosophical debate over the definition of death and many potential pitfalls that would threaten their expressed desire for uniformity.

One philosophical aspect to the debate that was unavoidable, however, was the question of formulation. Any recommended uniform law would have to engage with the question of the whole‐brain versus neocortical versus destructionist formulations of brain death. Here, the President's Commission deferred to the median position of whole‐brain death. The report echoed arguments from commentors like Roelofs, agreeing that, while neocortical death was a philosophically coherent position, the technical capabilities of the medical field were insufficient for it to be effectively translated into statutory language.^58^ In addition, they expressed concerns that neocortical death departed too radically from the traditional position of death, which could undermine support for the law.^59^ The report rejected the destructionist argument as well, pointing out that destruction of the cardiopulmonary system was not required to declare death by traditional cardiopulmonary criteria. The UDDA strongly affirms the whole‐brain position, stating that “irreversible cessation of all functions of the entire brain, including the brain stem,” is required for a declaration of neurologic death.^60^

The UDDA was not without detractors. In particular, Bernat emerged as a prominent critic, specifically of the drafters’ decision to avoid defining the relationship between the circulatory and brain death criteria. In contrast, a proposal published by Bernat, Charles Culver, and Bernard Gert reverses the formulation developed by Capron and Kass: instead of a standard circulatory criteria with a situational exception for the use of a brain death standard, the Bernat proposal, based on the philosophically coherent idea that all death is brain death, included an explicit carve out for the practical use of circulatory criteria as a standard for death.^61^ Bernat, Culver, and Gert argued that this was preferable to the UDDA because it made clear that death was a unitary phenomenon defined by the loss of the brain's ability to integrate the function of the organism and that, while cessation of heart and lung function could be a standard used to demonstrate cessation of brain function, it was not a sufficient standard for death.^62^ The Bernat proposal makes a philosophical argument—that all human death is brain death.

*Defining Death* suggested that a model statute, unlike the Kansas statute, must make clear that death was a unitary phenomenon, but it still provides two definitions.^63^ In its final section, which promotes the UDDA as an appropriate model statute, the report argues that a unified brain death statute such as the Bernat proposal, “breaks with tradition in a manner that appears to be unnecessary.”^64^ While Bernat et al. acknowledged that the UDDA would likely function in the majority of circumstances, they felt that the language of the act left some uncommon cases open to unacceptable ambiguity. “[A]ny standard of death,” they stated, “must be adequate in all cases, not merely the overwhelming number.”^65^ The report of the President's Commission, however, makes it clear that the UDDA itself was crafted, not to be a perfect standard of death, but, rather, an adequate standard of death that would be acceptable to the majority of stakeholder professional organizations. This meant that state legislatures could feel confident in adopting the UDDA because they no longer had numerous competing model statutes to sift through and, as a result, there would be improved legal uniformity.^66^ The President's Commission's report demonstrates that the UDDA prioritized uniformity over conceptual clarity in edge cases.

The UDDA built consensus around its legislation by deliberately not engaging with the underlying philosophical debates that surrounded brain death. As *Defining Death* makes clear, the UDDA was not built to accomplish a completely philosophically coherent definition of death; instead, it was designed to be good enough, and broadly acceptable enough, to accomplish the goal of legal uniformity. The UDDA was weakened in its applicability to fringe cases by its drafters’ prioritization of consensus and uniformity. As a result, the UDDA did not resolve the philosophical and legal confusion of the 1970s but merely elided that confusion. However, the UDDA's concessions to philosophical coherence worked—it was quickly and broadly adopted by state legislatures, replacing the patchwork of confusing state‐specific statutes with a largely nationally consistent definition of death. However, contemporary commentators such as Bernat expressed concern that what was “good enough” for the majority of cases might eventually prove insufficient given the rapidly evolving pace of technical‐medical capabilities.

## The Fault Lines of Consensus

In 2020, the Uniform Law Commission initiated an attempt to revise the UDDA. This effort was unsuccessful and was ultimately paused indefinitely in 2023.^67^ The whole‐brain‐death principle, which in the 1970s and 1980s was so critical as a median position, has been called into question because physicians cannot reliably test for all the functions of the entire brain.^68^ The concept of irreversibility has become problematic in the context of increasingly advanced resuscitation technology. Finally, there has been an increasing resistance to the concept of brain death from some patient families, which has resulted in a proliferation of legal cases challenging the concept of brain death itself.^69^ The failure of the revision process is indicative of the deep underlying divisions in stakeholders’ understandings of higher principles—principles that were never resolved by the UDDA, only elided in the interests of uniformity. The drafting committee of the Uniform Law Commission, in their announcement of a pause, stated that “we will continue to hope mid‐level principles will become apparent.”^70^ The fragile midlevel principles on which the 1980 UDDA was built are eroding, and new midlevel principles that would provide fruitful ground for revision have not emerged.

The wide gaps in philosophical and religious perspectives on brain death have not closed since 1980. On the contrary, they have deepened. The problem of uniformity remains a significant challenge. The report of the President's Commission made the case that an effective statutory definition of death need not take a philosophical stance. The Commission argued that a statutory definition that remains grounded in pure philosophical principles may be strong in theory but would in fact prove practically weak because consensus building around such a statute—a statute for the ivory tower, not the halls of the state house—would be difficult. The President's Commission acknowledged that the UDDA left gaps, but argued that those gaps were sufficiently narrow, and the benefits of wide applicability sufficiently great, to justify a less‐grounded statute. In response to the President's Commission, Bernat, Culver, and Gert argued that a definition of death should be sufficiently grounded in underlying principles to allow for applicability, not just in the majority of cases, but in all cases. The UDDA functioned effectively for over thirty years, but technological evolution suggests that Bernat and his collaborators’ concerns were warranted. The recent failed attempt to revise the UDDA demonstrates the major difficulty of creating a broadly acceptable statute—even one based on midlevel principles. Statutory revision of the UDDA will at some point become unavoidable, and a successful revision will require more thorough commitment to higher philosophical principles than seen in its predecessor.
